# Expression and Significance of Insulin Receptor Substrate 1 in Human Hepatocellular Carcinoma

**DOI:** 10.1155/2020/7174062

**Published:** 2020-07-10

**Authors:** Chun Gao, Hui Zhang, Wei-Shuo Zhang, Long Fang

**Affiliations:** ^1^Department of Gastroenterology, China-Japan Friendship Hospital, Ministry of Health, Beijing 100029, China; ^2^Department of Gastroenterology, Beijing Tiantan Hospital, Capital Medical University, Beijing 100050, China

## Abstract

**Background:**

Insulin receptor substrate 1 (IRS-1) is an important molecule of the insulin signal transduction pathway and has been associated with the occurrence and development of many tumors, including hepatocellular carcinoma (HCC). Our study was designed to determine the expression and significance of IRS-1 in human HCC.

**Methods:**

Two hundred and forty specimens were drawn from 140 patients, including 100 HCC tissues and 100 paracancerous (PC) tissues from 100 HCC patients, 20 liver cirrhosis (LC) tissues from 20 LC patients, and 20 chronic hepatitis (CH) tissues from 20 CH patients. Baseline and pathological characteristics were included, and the expression of IRS-1 was examined by immunohistochemical (IHC) staining. Binary logistic regression model calculation was used for multivariate analysis.

**Results:**

The total positive rates of IRS-1 expression were 41.0%, 17.0%, 15.0%, and 10.0% in HCC, PC, LC and CH tissues, respectively. IRS-1-positive signals were brown in color and located in the nucleus and cytoplasm. Compared with PC, LC, and CH tissues, a significantly increased expression was observed in human HCC tissues (*P* < 0.001, *P* = 0.028, and *P* = 0.008). Eight of the total 240 specimens had the strong immunostaining of IRS-1 expression, and all of them were HCC tissues. After control of the age, gender, and HBV and HCV infection, IRS-1 expression was independently associated with the diagnosis of HCC (OR 6.60, 95% CI 2.243-19.425, *P* = 0.001).

**Conclusions:**

Positive expression of IRS-1 in HCC was increased significantly and may play an important role in the occurrence and development of human HCC.

## 1. Introduction

Hepatocellular carcinoma (HCC) is the most prevalent malignant cancer of liver and is one of the major causes of tumor-related mortality, particularly in China [1–3]. The unfavorable prognoses can be observed in most HCC patients, although great progress has been made in the early diagnosis and HCC-associated treatment [4, 5]. Identification of causes and risk factors can facilitate early intervention and improve outcome, and the risk factors of HCC include hepatitis B virus (HBV), HCV infection, aflatoxin exposure, heavy alcoholic consumption, and liver cirrhosis [6, 7]. Unfortunately, the definitive risk factors have not been found in about 15-50% of HCC patients [6–8].

Diabetes mellitus (DM) has been confirmed as one of the important risk factors of HCC in the last decade [9, 10]. One systematic review and meta-analysis included 25 cohort studies and found that DM was associated with the increased risk of HCC and the summary relative risk (SRR) was 2.01 [10]. Another prospective cohort study which was conducted in Taiwan region of China utilized a representative sample of 800,000 participants and showed that the HCC incidence density increased at least twice in diabetic patients [11]. The conclusion was supported by many follow-up studies [12, 13].

Insulin receptor substrate 1 (IRS-1) is an important molecule of the insulin signal transduction pathway and plays multiple varieties of biological regulations in insulin metabolism [14, 15]. Recently, some studies showed that IRS-1 was associated with the occurrence and development of several different tumors, including HCC, lung cancer, breast cancer, pancreatic cancer, ovarian cancer, and renal cell carcinoma [16–19]. Tanaka et al. analyzed the IRS-1 gene expression in 22 human HCC tumors and adjacent noninvolved liver tissues and found that approximately 40% of them had enhanced (>200%) expression compared with paracancerous tissues [16]. Considering the limited studies and patient population, our research was designed to determine the expression and significance of IRS-1 in human HCC, compared with other liver diseases.

## 2. Patients and Methods

### 2.1. Study Patients

Two hundred and forty specimens were drawn from 140 patients, including 100 HCC tissues and 100 paracancerous (PC) tissues from 100 HCC patients, 20 liver cirrhosis (LC) tissues from 20 LC patients, and 20 chronic hepatitis (CH) tissues from 20 CH patients. These patients were treated at the China-Japan Friendship Hospital, Ministry of Health, and the Beijing Tiantan Hospital, Capital Medical University. Patients who followed these criteria were included: (1) those who had undergone surgical treatment because of tumor or other diseases and the surgical specimens could satisfy the need of scientific research and experiments; (2) those who had not been treated with any tumor-associated therapies before surgery; (3) HCC, LC, and chronic hepatitis were diagnosed by pathological examination; (4) those who had no presence of other malignancies, including lymphoma and leukemia; and (5) those who had no other severe disease, including heart, lung, brain, kidney, rheumatic, or allergic disorders.

Our research project followed strictly the principles of the Declaration of Helsinki, and it was approved by the Human Research Ethics Committee of China-Japan Friendship Hospital, Ministry of Health. Informed consent was obtained from all patients.

### 2.2. Specimen Preparation and Experimental Materials

All the acquired surgical specimens were fixed immediately in 4% paraformaldehyde for 24 hr and transported to the laboratory for subsequent processing. They proceeded to be dehydrated and cleared in gradient ethanol and xylene, finally embedded in paraffin blocks. For pathological diagnosis, these paraffin-embedded tissues were serially sectioned and stained with hematoxylin-eosin. For immunohistochemical staining, they were cut as 4-5 *μ*m thick and mounted on poly-lysine-coated slides.

Polyclonal antibody anti-HIRS-1 (No. SAB4300482-100UG), specially prepared for immunohistochemistry (IHC), was obtained from Sigma-Aldrich (Saint Louis, MO, USA). The original concentration was 1 mg/ml, and the final optimal diluted concentration was 1 : 200. The PV-6001/6002 PowerVision™ two-step histostaining reagent was a ready-to-use kit and purchased from Zhongshan Jinqiao Biotechnology Corporation (Beijing, China). Some routine reagents, including phosphate-buffered saline (PBS) buffer, citrate buffer, and Dako REAL™ EnVision™ Detection System, were also supplied by Zhongshan Jinqiao (Beijing, China). In addition, a microwave oven operating at a frequency of 2.45 GHz was used for antigen retrieval.

### 2.3. Observation of Relevant Indicators

Five relevant indicators were recorded, including their ages, sex, infection status of hepatitis B virus (HBV) and/or hepatitis C virus (HCV), and pathologic diagnosis of HCC or others. For subsequent analysis, the age was regrouped into two groups with the cutoff of 55 years old. Pathological characteristics of the 100 HCC patients were shown, including the TNM stage, clinical classification, grade of differentiation, presence or absence of tumor thrombus of veins, location of tumors, tumor number, and the maximum diameter of tumors. For the clinical classification, three types were found in these patients, including massive type, nodular type, and small-cancer type.

### 2.4. Immunohistochemical Staining

Immunohistochemical (IHC) staining was strictly carried out following the measure operation steps of the manufacturer's instructions. According to our previously published studies [20–22], microwave oven heat-induced antigen retrieval in immunostaining can replace the pretreatment with diluted 3% hydrogen peroxide in methanol. For every examination batch, three kinds of controls were set up, including one kind of positive control and two kinds of negative control. The paraffin sections for negative control were obtained from our previous experiments or by replacing the primary antibody with 0.9% sodium chloride solution. The paraffin sections for positive control were purchased from the Lab Vision or Maixin Biotechnology (Fuzhou, China).

IRS-1 expression was evaluated by two researchers independently, and if they obtained inconsistent results, consultation and discussion would be used. The extent of immunostaining was scored from grade 1 to grade 4: 1, 5-25%; 2, 25-50%, 3, 50-75%; and 4, 75-100%. The staining intensity was scored from grade 1 to grade 3: 1, mild; 2, moderate; and 3, strong. The final score was the sum of two numbers, and the expression grade was determined as 2-3, weak or mild; 4-5, moderate; and 6-7, strong.

### 2.5. Statistical Analysis

SPSS for Windows, version 21.0 (SPSS, Chicago, IL, USA) was used for statistical analysis. The continuous variables were described as mean ± standard deviation, and the categorical variables were presented as numbers and proportions. For univariate analysis, we used one-way ANOVA, Pearson chi-square tests, continuity correction chi-square tests, or Fisher's exact tests, respectively. For multivariate analysis, binary Logistic regression model calculation was used to determine the association of baseline characteristics with IRS-1 expression. Considering the relevant indicators and study population, 5 variables were entered, including age, sex, infection status of HBV and/or HCV, and pathologic diagnosis of HCC or others. The results were expressed as adjusted odds ratios (ORs) and their 95% confidence intervals (CIs).

## 3. Results

### 3.1. Baseline Characteristics of 140 Patients and 240 Specimens

Our study included 240 surgical specimens which were drawn from 140 patients, including 100 HCC tissues and 100 PC tissues from 100 HCC patients, 20 LC tissues from 20 LC patients, and 20 CH tissues from 20 CH patients.  Table 1 shows the baseline characteristics of 140 patients, and Table 2 demonstrates the pathological characteristics of 100 HCC patients. Among the 140 patients, 92 were male, 88 were diagnosed to have HBV infection, 11 patients were diagnosed to have HCV infection, and all the diagnoses were confirmed by pathological examinations. For the 5 baseline characteristics, no significant differences were found by univariate analysis (Table 1).

For the TNM stage of the 100 HCC patients, 63 were defined as Stage T1 and Stage T2; no patient was found to have lymph node metastases, and only 2 patients were identified as Stage M1. The mean maximum diameter of tumors was 7.09 ± 3.66 cm, and 17% patients were diagnosed to have tumor thrombus of veins (Table 2).

### 3.2. Expression of IRS-1 in Human HCC and Paracancerous Tissues

**Figure 1:**
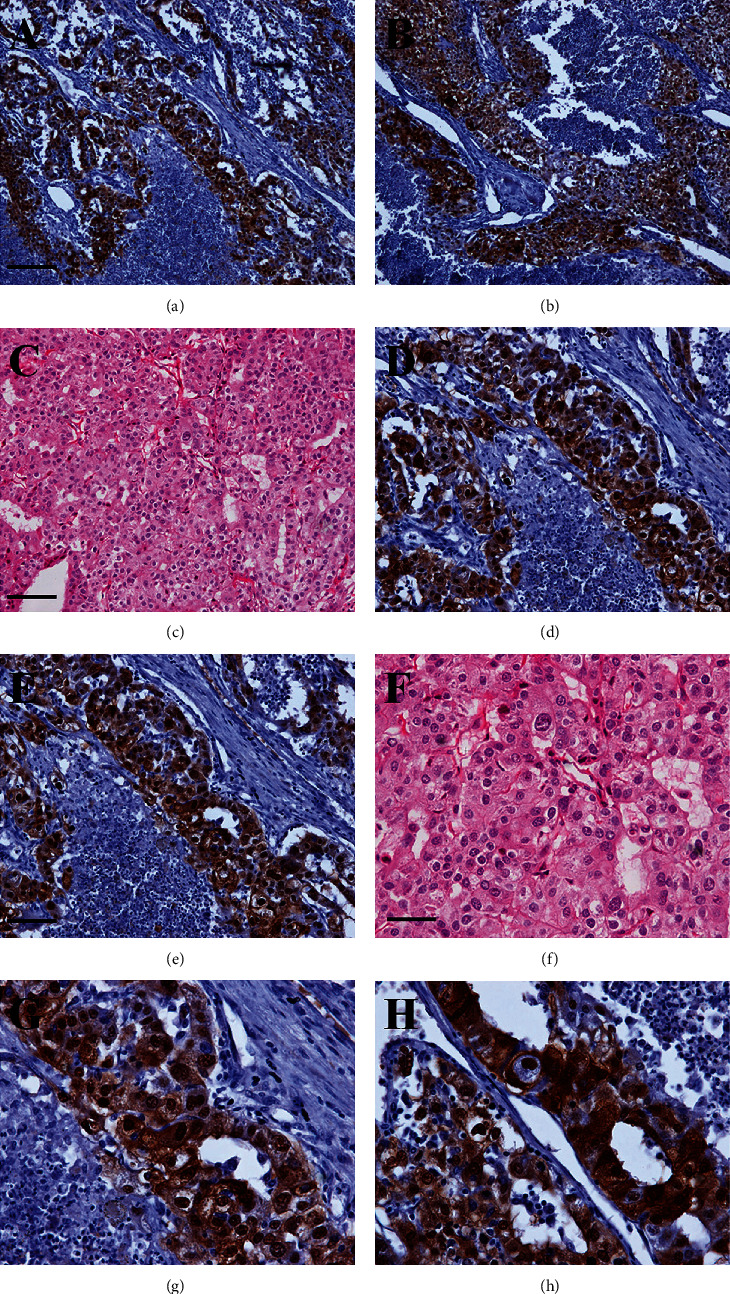
Expression of IRS-1 by immunohistochemical analysis in human hepatocellular carcinoma (HCC) tissues. (a-h) Immunohistochemical staining; (c, f) hematoxylin-eosin staining. Bars: a, b = 160 *μ*m; c − e = 80 *μ*m; f − h = 40 *μ*m.

Expression of IRS-1 was examined by IHC staining, and Table 3 shows the IRS-1 expression in human HCC tissues (Figure 1). Among the 100 HCC specimens, 41% was observed to have the positive expression of protein IRS-1. For the grade of immunostaining intensity, 15%, 18%, and 8% of patients were detected to have weak, moderate, and strong expression, respectively. The remaining 51 patients were negative.

**Figure 2:**
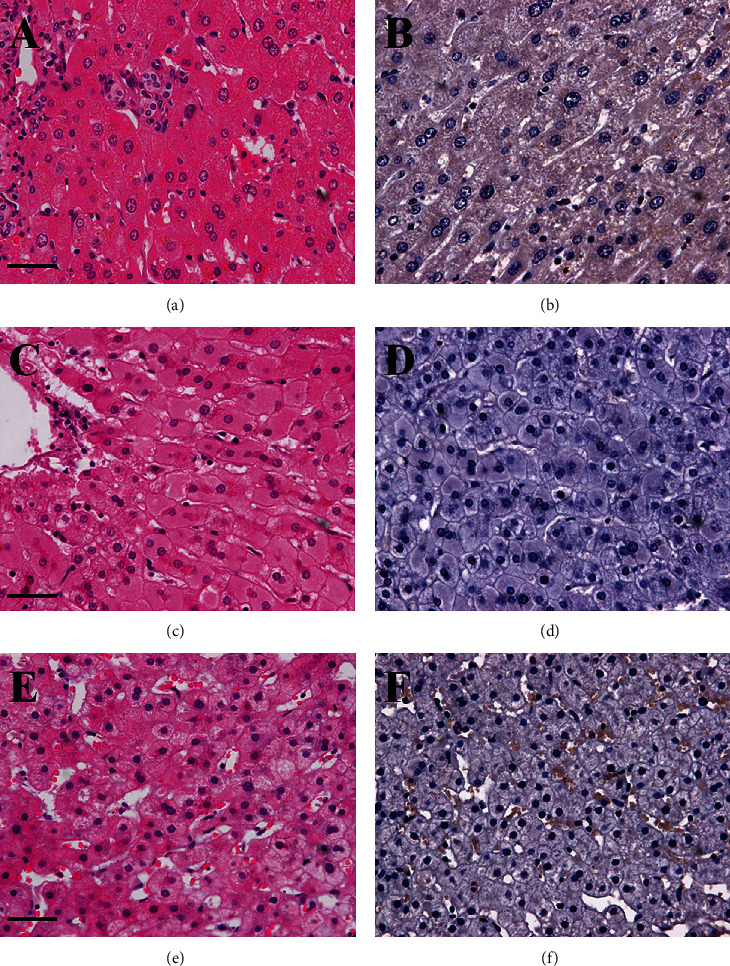
IRS-1 expression by immunohistochemistry in paracancerous tissues (a, b), liver cirrhosis (c, d), and chronic hepatitis tissues (e, f). (a, c, e) Hematoxylin-eosin staining; (b, d, f) immunohistochemical staining. Bars: a − f = 40 *μ*m.

The 100 paracancerous tissues specimens were drawn from the same group patients, and they had a one-to-one relationship with the 100 HCC samples. Figures 2(a) and 2(b) and Table 3 demonstrate the expression of IRS-1 in human PC specimens. We found that 17 patients had a positive expression of protein IRS-1. Eleven patients were weak positive, six patients were moderate, and no patient was strong expression.

### 3.3. Expression of IRS-1 in Liver Cirrhosis and Chronic Hepatitis Tissues

For the 20 LC patients and 20 CH patients, IRS-1-positive cells were observed in five patients (Figures 2(c)-2(f), Table 3). IRS-1 expression was weak in 2 (10.0%) LC patients and 2 (10.0%) CH patients, and moderate in one (5.0%) LC patient, and none (0.0%) of the CH patients. The strong expression of immunostaining was observed in none (0.0%) of the LC and CH patients. As demonstrated in Figures 1 and 2, the positive signals of IRS-1 expression were brown in color and located in the nucleus and cytoplasm. When the staining intensity was mild, they were observed mostly in the nucleus.

### 3.4. Expression of IRS-1: Increased Significantly in Human HCC

**Figure 3:**
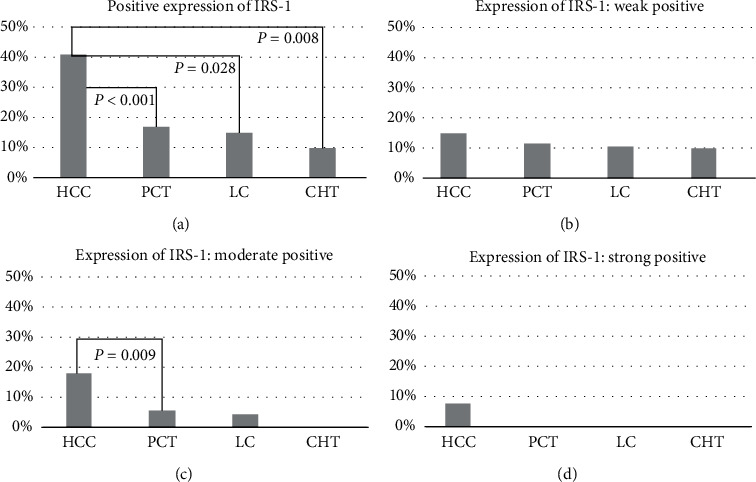
Proportions with the total positive expression (a), weak positive (b), moderate positive (c) and strong positive expression (d) of IRS-1 in four group tissue samples, including hepatocellular carcinoma (HCC), paracancerous tissues (PCT), liver cirrhosis (LC), and chronic hepatitis tissues (CHT). The *P* values were marked in the figure only when they were found as statistically significant different at *P* < 0.05.

The Pearson chi-square test was used to compare the expression intensity of protein IRS-1 in human HCC with other tissues. For the total positive rate of IRS-1 expression, a significantly increased level was observed in human HCC (Figure 3), compared with those in paracancerous tissues (PCT) (41/100, 41.0% *Vs.* 17/100, 17.0%, *P* < 0.001), LC (41/100, 41.0% *Vs.* 3/20, 15.0%, *P* = 0.028), and CH tissues (CHT) (41/100, 41.0% *Vs.* 2/20, 10.0%, *P* = 0.008). For the intercomparison of IRS-1 expression between PCT, LC, and CHT, no significant differences were found (*P* = 1.000, *P* = 0.655, and *P* = 1.000). Among the 140 patients, a moderate expression of IRS-1 was observed in 19 patients, including 18 HCC patients and only one LC patients. Eight of the 240 specimens had the strong immunostaining of IRS-1 expression, and all of them were HCC tissues.

### 3.5. Association of Baseline Characteristics with IRS-1 Expression: Logistic Regression

The 140 patients were redivided into two groups based on the expression status of protein IRS-1 to determine the association of baseline characteristics with IRS-1 expression (Table 4). Seventy-one patients were over 55 years of age, and 35.2% of them were positive expression of IRS-1. For the gender, 37.0% male and 25.0% female patients had the IRS-1-positive immunostaining. The positive signals of IRS-1 expression were observed in 29.5% of the patients with HBV infection and 18.2% of those with HCV infection. No statistical differences were found for these indicators.

Binary logistic regression model calculation was used to perform the multivariate analysis. Considering the results of univariate analysis and study population, five variables were entered, including the age, sex, infection status of HBV and/or HCV, and pathologic diagnosis of HCC or others. We found that significant differences were observed for the presence of HBV infection (OR 0.390, 95% CI 0.169-0.904, *P* = 0.028) and pathologic diagnosis of HCC (OR 6.60, 95% CI 2.243-19.425, *P* = 0.001).

## 4. Discussion

Diabetes has been identified as one risk factor for HCC, and IRS-1 is an important molecule of the insulin signal transduction pathway of DM. Based on these theoretical foundations, our study was designed to determine the expression and significance of IRS-1 in human HCC. PC, LC, and CH tissues were served as control subjects, and we found that the positive expression of IRS-1 in HCC was increased significantly. In addition, we observed that eight of the total 240 specimens had the strong signals of IRS-1-positive expression and all of them were HCC tissues. Moreover, multivariate analysis supported this conclusion and showed that IRS-1 was independently associated with the diagnosis of HCC and the value of adjusted OR was 6.60.

Our results indicated that increased IRS-1-positive expression may play an important role in the occurrence and development of human HCC. Some efforts have been made to gain these relatively reliable results. The first was that 200 HCC and paracancerous tissues were included in our examinations and they had a one-to-one relationship with each other. Such a comparison was much better than the operation in which HCC and PCT were drawn from different patients. The second was that 40 LC and CH patients were also served as the control subjects. Coincidentally, the pathological diagnosis of PCT was divided into two types, namely, LC and CH. Therefore, these two groups of specimens could be regarded as the controls either for HCC or for PCT.

Our conclusion that positive expression of IRS-1 was increased significantly in HCC was supported by some of the previously published studies [16, 23, 24]. In one study which was designed to determine the biological effects of IRS-1 overexpression in hepatocytes, Tanaka et al. completed the examination of 40 human HCC tumors and found that approximately 40% of them had a significantly increased expression of IRS-1 compared with paracancerous tissues [16]. In addition, the authors observed the significant relationship between the tumor size of HCC and the level of IRS-1 expression. Sakurai et al. used the diethylnitrosamine- (DEN-) induced HCC mice model and showed that the mRNA and protein levels of IRS-1 expression were upregulated in the HCC tumors, along with enhanced insulin signaling [23].

However, there is also a different voice, such as another research which was conducted to detect the expression of human IRS-1 and insulin signaling proteins under different pathophysiologic conditions of hepatic disease [25]. Thirty surgical specimens were processed in this study, including 10 HCC, 10 cirrhotic liver, and 10 of normal human liver. The positive relationship of IRS-1 expression between HCC and LC or normal human liver was not found, although the authors revealed that IRS-1 expression was increased significantly in cirrhosis compared to normal liver tissues [25]. For the seemingly contradictory results, the likely explanation was deduced to be associated with the very limited study population and the main purposes of these studies. In vivo and in vitro experiments were the main techniques in almost all of these researches.

Our study included 240 surgical specimens drawn from 140 patients and found that the IRS-1 expression was increased significantly in HCC. We also found that 33 of the 240 specimens were moderate or strong positive of IRS-1 expression, and 78.8% of the 33 specimens were HCC. Only eight were strong positive, and all of them were HCC. Considering that PCT can only be diagnosed as LC or CH in our study, the 140 non-HCC specimens were divided into two subgroups, namely, LC and CHT. For the IRS-1 expression, subgroup analysis was performed and no significantly statistical differences were observed. These further analyses supported our final conclusion.

Fortunately, some studies have investigated the molecular mechanisms of the effect of IRS-1 overexpression on the occurrence and development of HCC and their results supported our conclusion [16, 23, 26]. One study found that the IRS-1 expression was upregulated and insulin signaling was enhanced in the HCC tumors of the DEN-induced mice model [23]. The authors also demonstrated that IRS-1 was upregulated by Wnt/*β*-catenin signaling and associated with insulin-stimulated Akt activation [23]. Another research suggested that activation of the insulin (IN)/IRS-1/MAPK and the Wnt/*β*-catenin signaling cascades was associated with the development of HCC [26].

In addition, in our study, we found that the status of HBV infection was associated with the positive expression of protein IRS-1 (Table 4). The value of adjusted OR was 0.390, which indicated that the presence of HBV infection may be a protective factor for the IRS-1-positive expression. Considering the association of HBV infection with HCC, it theoretically may be a risk factor for IRS-1 expression. One study used the HBV-related double transgenic murine model and showed that overexpression of HBV X protein (HBx) and IRS-1 could stimulate cell proliferation in the liver [27]. The reason for our result was deduced to be associated with the limited number of patients diagnosed with HBV infection in our study. The definite reason needs to be clarified.

Some limitations should be acknowledged. The first was that all the patients were drawn from one medical center of China and all the specimens were obtained from the surgery operation. Therefore, bias selection could not be avoided and we set up some different kinds of controls to ensure the accuracy of the results. The second was that the expression was examined simply by IHC and more techniques were required.

In conclusion, our study found that IRS-1-positive expression in HCC was increased significantly and may play an important role in the development of human HCC. For a better understanding of the molecular mechanisms, more studies are required.

## Figures and Tables

**Table 1:** Baseline characteristics of patients^∗^.

Variables	HCC	LC	CH	*P* value
Total number, *n*	100	20	20	
Number of specimens, *n*	200	20	20	
HCC	100			
Paracancerous tissues	100			
Age, years				
Mean age	56.6 ± 10.6	54.0 ± 11.0	54.1 ± 10.3	0.427
≥55, *n* (%)	55 (55.0)	8 (40.0)	8 (40.0)	0.276
<55, *n* (%)	45 (45.0)	12 (60.0)	12 (60.0)	—
Sex				
Male, *n* (%)	69 (69.0)	13 (65.0)	10 (50.0)	0.262
Female, *n* (%)	31 (31.0)	7 (35.0)	10 (50.0)	—
Hepatitis virus				
HBsAg+, *n* (%)	66 (66.0)	11 (55.0)	9 (45.0)	0.173
Anti-HCV+, *n* (%)	7 (7.0)	1 (5.0)	1 (5.0)	0.909
Both HBV and HCV, *n* (%)	2 (2.0)	0 (0.0)	0 (0.0)	—
None, *n* (%)	25 (25.0)	8 (40.0)	10 (50.0)	0.054
Pathologic diagnosis				
HCC, *n* (%)	100 (100)	—	—	
Cirrhosis, *n* (%)	—	20 (100)	—	
Chronic hepatitis, *n* (%)	—	—	20 (100)	

CH: chronic hepatitis; HCC: hepatocellular carcinoma; LC: liver cirrhosis ^∗^Two hundred and forty specimens were drawn from 140 patients, including 100 HCC tissues and 100 paracancerous tissues from 100 HCC patients, 20 cirrhosis tissues from 20 LC patients, and 20 chronic hepatitis tissues from 20 CH patients.

**Table 2:** Pathological characteristics of 100 HCC patients.

Variables	100 HCC patients
T stage, *n* (%)	Stage T1-2, 63 (63.0)
	Stage T3-4, 37 (37.0)

N stage, *n* (%)	Stage N0, 100 (100.0)
	Stage N1, 0 (0.0)

M stage, *n* (%)	Stage M0, 96 (96.0)
	Stage M1, 2 (2.0)

Clinical classification, *n* (%)	Massive type, 54 (54.0)
	Nodular type, 33 (33.0)
	Small-cancer type, 13 (13.0)

Grade of differentiation, *n* (%)	Middle, 79 (79.0)
	High and low, 21 (21.0)

Tumor thrombus of veins, *n* (%)	Absence, 83 (83.0)
	Presence, 17 (17.0)

Location of tumors, *n* (%)	Left liver, 17 (17.0)
	Right liver, 74 (74.0)
	Left and right liver, 9 (9.0)

Tumor number, *n* (%)	One, 61 (61.0)
	Two or more, 39 (39.0)

Maximum diameter of tumors, cm	7.09 ± 3.66

**Table 3:** Expression of insulin receptor substrate 1 (IRS-1) in human HCC.

Tissues/protein	IRS-1 expression			
	Negative	Weak	Moderate	Strong
Tissues of detection				
HCC, *n* (%)	59 (59.0)	15 (15.0)	18 (18.0)	8 (8.0)
PCT, *n* (%)	83 (83.0)	11 (11.0)	6 (6.0)	0 (0.0)
LC, *n* (%)	17 (85.0)	2 (10.0)	1 (5.0)	0 (0.0)
CHT, *n* (%)	18 (90.0)	2 (10.0)	0 (0.0)	0 (0.0)
*P* value				
HCC *Vs.* PCT	<0.001^∗∗^	0.400	0.009^∗∗^	—
HCC *Vs.* LC	0.028^∗^	0.815	0.263	—
HCC *Vs.* CHT	0.008^∗∗^	0.815	—	—
PCT *Vs.* LC	1.000	1.000	1.000	—
PCT *Vs.* CHT	0.655	1.000	—	—
LC *Vs.* CHT	1.000	1.000	—	—

CHT: chronic hepatitis tissues; HCC: hepatocellular carcinoma; LC: liver cirrhosis; PCT: paracancerous tissues, ^∗^*P* < 0.05, ^∗∗^*P* < 0.01.

**Table 4:** Univariate and multivariate analyses for the association of baseline characteristics with IRS-1 expression in total 140 patients.

Variables	IRS-1 expression		Univariate	Multivariate analysis^†^		
	Positive	Negative	*P* value^∗^	Adjusted OR	95% CI	*P* value
Age (years), *n* (%)						
≥55	25 (35.2)	46 (64.8)	0.547	1.121	0.509-2.470	0.776
<55	21 (30.4)	48 (69.6)	—			
Sex, *n* (%)						
Male	34 (37.0)	58 (63.0)	0.153	1.818	0.778-4.247	0.167
Female	12 (25.0)	36 (75.0)	—			
HBV, *n* (%)						
Positive	26 (29.5)	62 (70.5)	0.278	0.390	0.169-0.904	0.028^∗^
Negative	20 (38.5)	32 (61.5)	—			
HCV, *n* (%)						
Positive	2 (18.2)	9 (81.8)	0.456	0.215	0.040-1.160	0.074
Negative	44 (34.1)	85 (65.9)	—			
HCC patients, *n* (%)						
HCC	41 (41.0)	59 (59.0)	0.001^∗∗^	6.60	2.243-19.425	0.001^∗∗^
Non-HCC	5 (12.5)	35 (87.5)	—			

CI: confidence interval; HBV: hepatitis B virus; HCC: hepatocellular carcinoma; HCV; hepatitis C virus; OR: odds ratio, ^∗^*P* < 0.05, ^∗∗^*P* < 0.01, ^**†**^In our multivariate analysis, 5 variables were entered, including age which was regrouped into two groups with the cutoff of 55 years old, sex, infection status of HBV, infection status of HCV, and pathologic diagnosis of HCC or others.

## Data Availability

The data used to support the findings of this study are available from the corresponding author upon reasonable request.
